# Methodologies for Detoxifying Bivalves from Marine Paralytic Shellfish Toxins

**DOI:** 10.3390/md23100398

**Published:** 2025-10-12

**Authors:** Adewale Aderogba, Joana F. Leal, Maria L. S. Cristiano

**Affiliations:** 1 Department of Chemistry and Pharmacy, Faculty of Sciences and Technology, Gambelas Campus, University of Algarve, 8005-139 Faro, Portugal; adewaleaderogba2020@gmail.com (A.A.); mcristi@ualg.pt (M.L.S.C.); 2 Centro de Ciências do Mar do Algarve (CCMAR/CIMAR LA), Campus de Gambelas, Universidade do Algarve, 8005-139 Faro, Portugal

**Keywords:** paralytic shellfish poisoning, bivalves, removal, detoxification, marine system, depuration, adsorbents, photodegradation, AOPs

## Abstract

The marine environment emerges as a key provider of food and sustainable products. However, these benefits are accompanied by numerous challenges owing to harmful algal blooms (HAB) and their associated biotoxins, which accumulate in organisms, like bivalves, threatening seafood quality. Among the various biotoxins, paralytic shellfish toxins (PST), the causative agents of paralytic shellfish poisoning (PSP), are among the most potent, lethal, and frequently reported instances of human intoxication. Removing PST from marine system is particularly challenging because of their hydrophilicity, susceptibility to biotransformation and the potential influence of other substances naturally present in the environment. Although there are several methods applied to mitigate HAB, to the best of our knowledge there are no proven effective methods for removing PST in marine environments. Consequently, there is a need to develop efficient removal technologies, especially envisaging fast, environmentally safe, inexpensive, and readily available solutions. Having examined several proposed methods for removing PST (e.g., thermal and industrial procedures, adsorption using different materials, photodegradation, AOPs) and comparing their efficacy, this study aims to streamline the current knowledge on PST removal, identify knowledge gaps, and provide valuable insights for researchers, environmental managers, and policymakers engaged in mitigating the risks associated with PST.

## 1. Introduction

The excessive growth of algae species often leads to phytoplankton proliferation in marine ecosystems, producing highly potent natural toxins, called phycotoxins or marine biotoxins, through a process known as harmful algae bloom (HAB). These biotoxins bioaccumulate through the food chain in aquatic species such as bivalves, crabs, and pufferfish, causing toxic effects in certain marine organisms and humans, when ingested. Their toxicity varies depending on the type of toxin and the amount ingested; at high doses, they are highly toxic and may lead to death [1]. In general, symptoms in humans may differ from severe gastrointestinal intoxication, with diarrhea, nausea, vomiting, and abdominal cramps, to neurological disorders such as ataxia, dizziness, partial paralysis, and respiratory distress [2]. As a preventive measure, if toxin levels surpass the established safety limits in a given area, prohibition of harvesting and complete closure of that area is the present regulatory approach. Although this approach can prevent human exposure, it often leads to socio-economic problems, such as high capital loss by aquaculture farmers and industries, scarcity of seafood, and negative impacts on tourism and recreational activities [3,4]. Among the main syndromes associated with marine HAB are paralytic shellfish poisoning—PSP, diarrheal shellfish poisoning—DSP, amnesic shellfish poisoning—ASP, azaspiracid poisoning—AZP, and neurotoxic shellfish poisoning—NSP [3,5]. Details on the properties and effects of each group of toxins are not the focus of this work and can be found in the recent literature [1,6,7,8,9]. This work will focus on the group of toxins that cause PSP because it is the one with the greatest lethal potential for humans and one of the most prevalent worldwide [10]. For instance, among the five most common toxic HAB syndromes (PSP, DSP, ASP, AZP and NSP), a maximum of events was reported in 2020 for PSP, corresponding to approximately 87% of the total worldwide (364/417 reports) [11].

**Figure 1:**
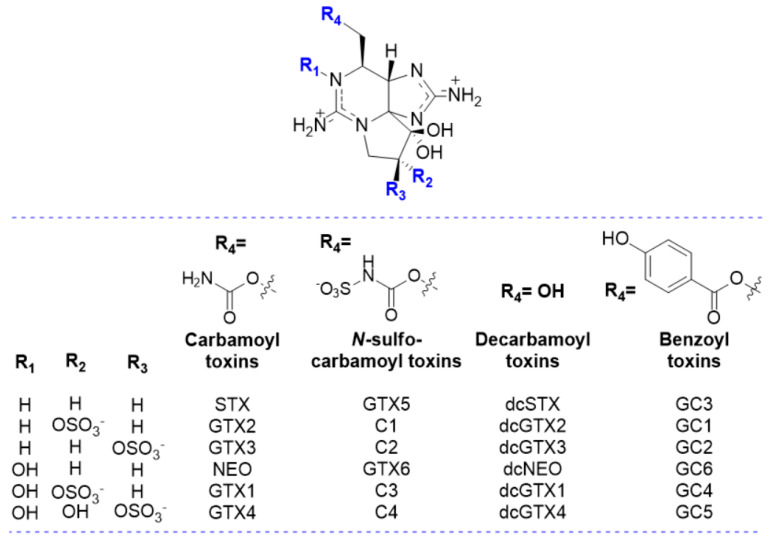
Structure representation of the main groups of Paralytic Shellfish Toxins (PST).

PSP toxins or Paralytic Shellfish Toxins (PST), also known as saxitoxins (STXs), comprise more than 50 derivatives of the parent compound, saxitoxin (STX) [1], one of the most potent analogues of the class, named after the toxic butter clam *Saxidomus giganteus*, from which it was first isolated [12]. The PST sub-groups are defined according to their characteristics, based on the substituent group in R_4_ (Figure 1). Most toxins belong to one of the subgroups, namely the carbamoyl (R_4_ = -OCONH_2_), *N*-sulfocarbamoyl (R_4_ = -OCONHSO_3_^−^), decarbamoyl (R_4_ = -OH), benzoyl (R_4_ = -OCOC_6_H_4_OH) and deoxydecarbomyl (R_4_ = -H) subgroups. Each substituent group affects the binding affinity to the receptor by steric hindrance, net charge, and/or polarity [13]. In addition, newly discovered analogues were unravelled, known as the M-series toxins [14,15,16], which are mainly distinguished by the presence of one or two -OH groups in R_2_/R_3_ (Figure 1).

Owing to the challenges associated with forecasting HAB and the severity of their intoxication, numerous countries have established regulatory thresholds for major marine toxins to safeguard public health. However, significant disparities exist in regulatory frameworks across different nations or regions. Developed regions, including the European Union, the United States, Canada, and Japan, enforce stringent monitoring programs and a maximum threshold for PSP toxicity in the edible tissues of aquatic organisms of 800 μg STX equivalents (eq). kg^−1^ shellfish meat [13,17,18]. On the other hand, regions in Africa, Southeast Asia, and Latin America often lack infrastructure, enforcement, or updated safety thresholds for PSP toxins. This regulatory gap increases the risk of human intoxication and hampers the international seafood trade due to inconsistent safety standards. Also, it could contribute to an inequality of opportunities for the development of detoxification methods.

The accumulation of PST in bivalves is a multifaceted phenomenon that is affected by several factors, including species-specific variations in feeding habits, rates of toxin absorption and elimination, biotransformation and metabolic functions. For instance, most mussels can eliminate PST faster than scallops because of their higher depuration rates [19].

Numerous techniques have been reviewed [20,21,22,23,24] and tested to contain PST-producing HAB and/or to remove PST in aquatic environments. Among the various studies, adsorption using different type of materials [25,26,27,28,29,30,31,32], biodegradation [33,34], nanofiltration [35], photodegradation [36,37], control feeding with non-toxic diets [38,39,40], industrial and thermal procedures [41,42,43], chlorination [44,45] and advanced oxidation processes (AOPs) [46,47] stand out. In the context of detoxification of marine ecosystems, two main limitations encompass the vast majority of the aforementioned studies: they are conducted in freshwater or ultrapure water (i) and they only use standards or toxin-producing cell cultures (ii). It is known that several natural factors, such as the presence and quantity of salts, dissolved and particulate organic matter, and other contaminants, affect the rates of processes like adsorption, photodegradation, or AOPs [29,48,49,50,51]. Failure to consider these factors when developing experiments limits their applicability to the marine aquatic environment. Furthermore, it is also known that the biotransformations that occur in organisms, particularly in bivalve molluscs, are different from those in microalgae [1] and are in no way comparable to the individual evaluation of standards. In fact, several studies [29,52,53,54,55] have evidenced differences in the toxin profiles of bivalves and the toxin-producing microalgae they ingest. This means that the methods proving to be efficient in aqueous solutions spiked with toxins, or in toxic microalgae cultures, may have their effectiveness altered in studies using bivalves, since the target or more abundant toxins may no longer be the same. Also, it is important to keep in mind that mitigating PST-producing algae is different from acting on toxins, since in the first case we are talking about algal cells, while in the second one we are talking about molecules that are released from the intracellular environment of the producing algal cells after their lysis. It is very difficult to prevent bivalves and other organisms from ingesting toxins naturally present in the water column. Therefore, the question that arises is: which of the techniques applied to HAB or tested in (fresh)water solutions can effectively be applied to the detoxification of bivalves to allow their safe consumption?

Through a comprehensive examination of current progress in mitigating PST, this review aims to provide valuable insights for researchers, environmental managers and policymakers engaged in mitigating the risks associated with PST. Its distinctive contribution to the field lies on the presentation a more in-depth approach on PST of marine origin, rather than a generalized view of several groups of toxins, and by delving into the methods applied to the detoxification of bivalves, focusing on a solution-based approach after toxin ingestion by bivalves. It also highlights the limitations and potentialities of the main techniques applied to bivalve detoxification, as well as some recommendations for further improvements.

## 2. PST Removal Methods

**Figure 2:**
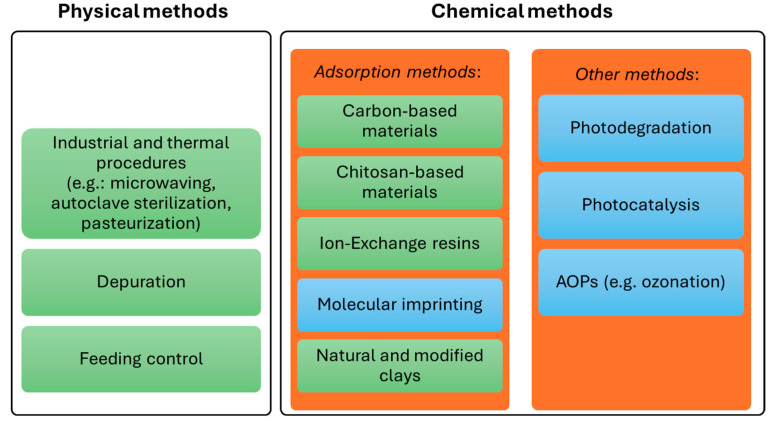
Physical and chemical methods described in the literature that are applied to PST degradation/removal. The methods highlighted in green have already been tested on bivalves, while those highlighted in blue have only been tested in aqueous solution.

Although marine aquatic systems frequently experience PST, no adequate and legally allowed methods exist to eliminate them from bivalves. This is probably due to their low efficiency in quickly reducing toxicity below the reference limit, or to regulatory concerns regarding their environmental impact. Various physical and chemical procedures have been tested to remove PST from marine systems (bivalves and water) or decrease their toxicity. They are summarized in Figure 2 and detailed below.

### 2.1. Physical Methods

#### 2.1.1. Industrial and Thermal Procedures

The European Commission approved in 1996 the thermal procedure for detoxification of a specific species of bivalves, the cockle *Acanthocardia tuberculata*, following the discovery that PST could be reduced to levels below the regulatory threshold through appropriate heat treatment [56]. In addition to being restricted to a single species of bivalves and only applicable to concentrations below 3000 μg STX diHCl equiv/kg, this method requires cooking the bivalves at high temperatures (95–98 °C) and autoclaving at a minimum temperature of 116 °C. More recently, some authors [42] have proposed a modification to this method, extending its implementation to mussels, clams, and scallops. In this procedure, the separation between edible and non-edible parts was omitted for mussels and clams. Additionally, pasteurization at 90 °C for 10 min was suggested as a possible alternative to autoclave sterilization at 116 °C for 51 min. The results revealed that this can be an efficient procedure to reduce toxicity in *Mytilus galloprovincialis*, *Ruditapes philippinarum* and *Pecten maximus*, but only for concentrations up to 5300 μg STX diHCl equiv/kg. However, to our knowledge, this method has not been legally recognized, so it cannot be implemented as a detoxification method. The same authors [42] suggested that in situations where PST concentrations in bivalves are not very high, applying the “detoxification procedure” could be unnecessary because regular canning or pasteurization steps appear to be satisfactory to reduce the toxicity levels. Nevertheless, the authors recommended that detoxification procedures be applied in all cases because mussel detoxification depends on several factors, such as the initial concentration, exposure time to toxins, and seawater conditions.

Recently, authors [41] investigated the degradation of PST from mussels using five thermal processes: steaming, boiling, frying, water-bath heating, and microwaving. Despite boiling, steaming, and microwaving decrease the toxicity in *M. edulis* tissues, the microwave heating procedure was the best, successfully destroying toxins at optimal power levels (420 W), reaching 99.51% of removal in tissues. Other methods, such as steaming, boiling, and water bath, remove toxins into the cooking liquor, while the frying procedure only concentrates the toxin, instead of reducing its concentration, as proved by the higher concentrations recorded compared to the control. The authors warn that consumption of cooking liquids resulting from steaming and boiling may be unsafe and may even cause poisoning, as the release of toxins from tissues into the liquid has been observed during the cooking process. Although partially effective in reducing toxicity to regulatory limits, the energy costs associated with these treatments and system adaptation to their implementation could also discredit the adoption of this methodology. Given the species specificities, further studies should be conducted considering other bivalves. Noteworthy, some studies revealed that consumers usually prefer fresh or preserved seafood (e.g., oysters) over processed alternatives [57]. This preference could limit the acceptance of detoxified bivalves by these methods and alter their marketability.

#### 2.1.2. Depuration and Feeding Control

Bivalve depuration involves placing the bivalves in clean flowing seawater under balanced conditions of salinity, temperature, and dissolved oxygen, so that the animals resume normal pumping activity and thereby expel contaminants from their gills and intestinal tract over time [58]. Depuration in tanks cannot yet be considered a viable means for removing biotoxin contamination to safe levels, especially as the depuration rates vary with the toxin and bivalve species and the process may take several days or months to achieve a reasonable removal rate. Some authors have reported that the presence of food in the environment can accelerate the purification of mussels [39] and oysters [38]. Other studies revealed that food deprivation for six weeks can promote mussel purification [59]. However, this approach for a long period of time is likely to compromise the quality of mussel meat and, consequently, its marketability. That said, further studies, encompassing more species, could be important to understand the role of non-toxic food in the purification rate.

### 2.2. Chemical Methods

The removal of PST from bivalves is instrumental to ensure food safety and public health. Various materials have been explored for their effectiveness in adsorbing PST, with emphasis on carbon-based and chitosan-based materials. Approaches involving the use of ion-exchange resins and modified clays have also been considered to accelerate the PST removal from bivalves. Additionally, the molecular imprinting technique and some advanced oxidation processes (AOPs) have been reported as potential methods, but only studies in aqueous solution are known so far.

#### 2.2.1. Carbon-Based Materials

To our knowledge, only one study reports the detoxification of bivalves in seawater, using activated charcoal. In this study, conducted in mussels and scallops, the authors highlighted a 68% and 25% decrease in the toxicity, respectively, after 72 h of contact [55]. They assessed the removal of PST from bivalve using eight different activated carbons, among which wood-based activated carbon (WAC) exhibited a maximum PST adsorption ratio of 65%, decreasing the toxicity of mussels by 41% after 1 day, and by 68% after 3 days of treatment. However, when applied to scallops, WAC only achieved a detoxification efficiency of 25%, after 3 days. This difference was ascribed to the species-specific accumulation and elimination rates of PST, which is faster for mussels than for scallops [19,60]. In fact, PST can be retained for over a year in scallops *Placopecten magellanicus*, due to their slow depuration rate [61,62]. Aiming to improve results, the authors suggested complementing the treatment with a diet of non-toxic algae, in line with the “feeding control” approach presented above [55].

It is worth noting that, unless the adsorbent material particles are small enough for the bivalve to absorb, the kinetics of toxin removal from the bivalve will be slower than if the toxins were directly dissolved in water. This is because, when dissolved in water, the interaction of toxins with the adsorbent is more direct than when they are in living tissue (bivalve). Nevertheless, the knowledge gathered from studies with toxin solutions may be relevant for optimizing detoxification in bivalves, namely:

- The removal of saxitoxin (STX) from water is pH-dependent and seems to be inhibited by the presence of natural organic matter (NOM) at neutral pH [28]. This inhibition may be explained either by the occupation or blocking of the pores of AC, or by preferential interaction/competition for the active sites available and electrostatic interactions with PST [28,63,64].
- STX adsorption by biochar could involve chemical and physical interactions, hydrogen bonding, Van der Waals forces, and pore filling [28,65]. Some of these mechanisms are favoured by the presence of acidic functional groups, such as carboxylic acid moieties, that have a high affinity for interacting with the polar guanidium groups of the STX molecules [32,65].
- The largest volume of mesopores seems to favour the adsorption of STX [63]. However, the same authors, who also tested dcSTX adsorption, did not observe any improvement in dcSTX adsorption capacity in the presence of more mesopores. This interesting observation could be explained by the lower steric hindrance associated with the presence of the -OH group at R4 in dcSTX, rather than the -OCONH2 group in STX (Figure 1). These findings indicate that the adsorption of different PST analogues will differ depending on their molecular structure.

Advancements in nanotechnology have positioned nanoparticles as promising tools for enhancing pollutant treatment and improving detoxification efficiency. Gonzalez-Jartin et al. [31] proposed a multicore magnetite coated with carbon nanoparticles (m-Fe_3_O_4_@C) and mesoporous silica nanoparticles (mesoporous-Si@Fe_3_O_4_) to remove marine toxins and cyanotoxins. The authors tested first the carbon and mesoporous silica particles in aqueous solutions (milli-Q water), reporting that mesoporous-Si@Fe_3_O_4_ particles (125 mg/L) did not show effectivity at removing PST after 60 min of incubation, while a reduction of around 40–45% was observed for STX, dcSTX and NEO when using m-Fe_3_O_4_@C particles. In subsequent studies the authors doubled the concentration of nanoparticles (250 mg/L) and studied the toxins by subgroup, suggesting that there is no competition between the different subgroups for sorption sites and that the adsorption capacity is greater at higher concentrations. Under these conditions, the maximum reduction in concentration was observed for STX, of 72% with 250 mg/L of m-Fe_3_O_4_@C, compared to 44% with 125 mg/L of m-Fe_3_O_4_@C, after the same incubation period. The authors also tested toxins produced by *G. catenatum*, namely C1, C2, dcSTX, dcNEO, GTX3, GTX5, and GTX6, to which STX (standard) was added by spike (20 µL/L). Among these toxins, STX, dcSTX, and dcNEO were the most susceptible to adsorption by carbon nanoparticles (m-Fe_3_O_4_@C), in keeping with the trend already observed in aqueous solution. While for STX the percentage of reduction is similar in aqueous solution and in cultures (approximately 44%), for dcSTX and dcNEO, the reduction in concentration was smaller (slightly above 20 and 30%, respectively). One of the important advantages of this type of nanoparticle is that they are easier to remove from systems due to their magnetic properties, enhancing the recycling and reuse potential of the method.

#### 2.2.2. Chitosan-Based Materials

Chitosan and its derivatives have emerged as promising candidates for removing toxins from bivalves. Researchers [66] have shown that combining carboxyl methyl chitosan (CMC, 50 mg/L) and *Platymonas subcordiformis* (PS) significantly improves the biotransformation and detoxification of PST in oyster *Ostrea rivularis*, compared to a starved control group. These authors observed that about 79% of the total toxicity was associated with the viscera, where a decrease below the legal limit has been achieved after 13 days. While on days 1 and 3 the presence of CMC with PS seems to accelerate the detoxification, compared to PS-only feeding, on the remaining days no significant differences were observed between both conditions. These results seem to suggest that the potential effect of an adsorbent (more localized and superficial action) will be predominant in the short term. In contrast, the effect of feeding seems to prevail in the medium and long term, since it directly impacts the metabolism of the bivalve [67]. Other authors [68] investigated three different depuration conditions to detoxify *Ostrea rivularis* Gould from PSP toxins, for 7 days. The faster depuration (~96%) was attained using the combination of chitosan with *Chlorella* spp., versus using only chitosan (~88%) or using only *Chlorella* spp. (~71%) [68]. The final toxicity reported was below the limit in all conditions, but the use of a blank control (no chitosan or food) to directly compare the results has not been mentioned. In contrast, Tobke et al. [69] did not observe a significant decrease in the overall toxicity, comparatively to controls, when assessing the PSP toxins depuration from mussels *M. chilensis* for 20 days, without adding food [69]. Both studies [68,69] have some limitations in experimental design, however there are three aspects that may contribute to the differences. Firstly, the different bivalve species, with different filtration rates (oysters vs. mussels). Then, in the first experiment non-toxic food was provided, unlike in the second one. Lastly, while Tobke et al. [69] reports the use of medium molecular weight chitosan, nothing is mentioned regarding the molecular weight range of chitosan used by Xie et al. [68]. Furthermore, the chitosan used in the studies has different origins, which could mean different characteristics, namely the molecular size, and consequent differentiated interaction with toxins. Thus, more details would be needed for a correct comparison of results. Another chitosan derivative, silica-malic acid chitosan hydrogel (SiO_2_-MA-CS), was applied to contaminated bay scallops, for five days. About 18% and 57% decrease in toxicity was observed in the hepatopancreas and kidneys, respectively [70]. Although the overall toxicity was above the regulatory limit after 5 days, the reported decrease in toxicity was approximately 55% (initial toxicity = 6766.70 μg STX eq kg ^−1^).

#### 2.2.3. Ion Exchange Resins and Molecular Imprinting

PST’s molecular structure and charge nature are other factors that have been explored when selecting a detoxification method. Based on these properties, cation exchange resins (H^+^ and Na^+^ forms) were investigated for removing PST from the algae culture of *Gymnodinium catenatum* and live mussels *(Mytilus edulis)* [29]. An 80% decrease in overall toxicity was reported after 48 h in vitro, but not in live mussels (*M. edulis*). The authors identified several reasons for the low efficiency, such as the competition of toxins with various natural substances (e.g., salts, organic matter) for the same binding sites, the blocking of pores due to interactions between molecules, and/or difficulties in resin absorption by mussels [29]. Interestingly, the authors observed a preferential removal of some toxins (e.g., dcSTX) over others (e.g., C1&2, GTX6), corroborating the hypothesis that molecular structure and overall charge play a determining role in the process.

In addition, another approach tested only in aqueous solution revealed that the molecular imprinting technique can be useful in the development of polymers capable of adsorbing PST [26]. The authors demonstrated that the use of template molecules together with functional monomers (*p*-styrene sulfonic acid) can enhance the adsorption of PST. From their study, the use of TBTA (4-(tributylammoniummethyl)-benzyltributylammonium chloride) as a template molecule appears to be more favourable than the use of BTAB (4,4′-bis(tributylammoniummethyl chloride)biphenyl). The dcSTX adsorption rates reported were approximately 84% or 47%, for polymers containing TBTA or BTAB as template molecules, respectively. The different performance of these polymers was attributed to the greater similarity in the distance between the ionic groups of TBTA (alkyl ammonium groups) and the guanidium groups of PST, in contrast to the greater distance between the positively charged groups in BTAB [26]. It may be interesting to test this principle in saltwater, covering more toxins, to evaluate the effect of the presence of salts on the interaction of polymers with toxins.

#### 2.2.4. Natural and Modified Clays

Natural mineral clays possess specific surface chemical properties, such as cation exchange capacity and adsorptive affinity for several compounds. This has led to investigations into their potential use as alternative materials for treating heavy metals and organic pollutants [71,72]. Clays are suitable for this purpose because they are naturally non-polluting, inexpensive, and readily available in enormous quantities [72].

Few studies have applied clays to PST removal. For example, Burns et al. [30] tested different clays (Na-montmorillonite, Ca-montmorillonite, kaolinite, well-crystallized kaolin, and poorly crystallized kaolin) and three different natural sediments for the adsorption of STX. The experiments were carried out in aqueous solutions (freshwater and synthetic saltwater) for 12 h. Among all adsorbents, they observed a higher adsorption by Ca-montmorillonite. Also, a positive linear correlation between the Freundlich constant (K_F_) (adsorption affinity) and the cation-exchange capacity (CEC) of different clays was reported, suggesting an ion-exchange mechanism for STX adsorption. Interestingly, they also observed higher K_F_ in freshwater than in seawater (~10^4^ μmol/kg and 10^3^ μmol/kg, respectively), concluding that the greater ionic strength of seawater may have an inhibitory effect on adsorption.

Other researchers [73] used modified clay based on polyaluminum chloride (PACl) to modify Kaolin (1:5) to treat a toxic bloom of *Alexandrium tamarense* and assessed differences in PST caused by the application of this modified clay. Although a decrease in the total PST concentration was observed, the experiment took several days (from day 25 to day 80). This makes its application in real systems (e.g., industry) technically and financially unfeasible, since it is an excessively time-consuming process. A polyaluminium chloride-modified clay (PAC-MC) was also considered by other authors that carried out their studies using live bivalves, namely the bay scallop *Argopecten irradians* [74]. After 3 h of exposure to toxic algae (*A. tamarense*), the authors reported higher toxicity levels in the control, in which modified clay had not been added, than in the groups containing 0.1 and 0.5 g/L of modified clay. This may be explained by the flocculation of toxic algae cells caused by the presence of modified clay, as has been reported by several authors [73,75,76,77]. In this way, there is a reduction in the food filtered by the bivalve, consequently decreasing the intake and accumulation of toxins in the living organism.

In a more recent study [78], a potassium peroxymonosulfate modified clay (PMPS-MC) was prepared by modifying the kaolin with potassium peroxymonosulfate (PMPS). This system leverages the flocculation properties of modified clay and the oxidizing capabilities of PMPS. Thus, PMPS-MC was employed to mitigate the harmful effects of *A. pacificum* blooms through the degradation of PST and removal of algae cells in a 48 h experiment. Different behaviours and efficiencies of degradation and removal were observed in the short (15 min), medium (9 h), and long term (48 h), and in the intra- and extracellular environment, suggesting different mechanisms at different stages and conditions. Exposure to 10 mg/L PMPS-MC resulted in a significant reduction in extracellular PST, dropping below detectable levels within 15 min, which corresponds to a 91% decrease in content and a 93% reduction in toxicity. Although a re-secretion was observed after 3 h, the levels remained lower than those in the controls. Additionally, PMPS-MC treatment significantly reduced intracellular PST, showing a 14–31% decrease within 15 min and consistently staying below control levels over 48 h, ultimately reaching about 54% of the control values. Also, this treatment seems to facilitate the conversion of more toxic PST analogues (GTX1&4) into less harmful forms (GTX2&3, C1&2), potentiating the reduction in toxicity.

The above-mentioned studies reveal the potential of modified clay composites as adsorbents of PST. Despite clay’s natural origin, it is essential to investigate its impact on marine life, along with that of any modifying substances, before implementing it. To promote the adoption of modified clay, it is also imperative to advance research on its environmental suitability, in situ performance, reusability, and economic viability.

#### 2.2.5. Photodegradation and Photocatalysis

Following the principle of the interval immobilization technique above described, Tominaga et al. [36] prepared two types of hybrids photocatalysts, both involving TiO_2_ particles. Following a contact of 10 min without light exposure, the suspension containing dcSTX was exposed to UV light at 254 nm for 40 min, and a sharp decline in dcSTX concentration was observed after 20 min. This study could open promising prospects for the photodegradation of PST, but development towards implementation must consider specificities closer to real-world conditions, for example, developing studies with more toxins in saltwater/seawater. In fact, a recent study [37] evaluated the photodegradation of other PST, namely STX, GTX2&3, and C1&2, in deionized water, at pH of 6 and 8, and the effect of dissolved organic matter (DOM) on the process. The authors highlighted that the toxins did not undergo direct photodegradation at any pH but noted that STX and GTX2&3 underwent DOM-sensitized photodegradation at pH 8, suggesting an electron-transfer mechanism between saxitoxin and ^3^DOM* (triplet state of dissolved organic matter). From our perspective, and assuming a system with controlled conditions, this technique would always have to be coupled with another prior technique that promotes the excretion of toxins from within the bivalves. Applying radiation to the bivalves would not be efficient, given their shell protection. Photodegradation will certainly be more efficient in aqueous solutions, when the toxins are dissolved in the medium. However, to the best of our knowledge no studies were conducted on PST photodegradation in saltwater.

#### 2.2.6. Advanced Oxidation Processes (AOPs)

As interest grows in finding suitable techniques to eliminate toxins from marine environments, some AOPs have emerged. AOPs utilize highly reactive radicals, such as hydroxyl, hydroperoxyl, superoxide, and sulphate, to partially or fully decompose emerging contaminants [79]. These technologies can reduce the dependence on harmful solvents or other chemical agents, thus aligning with environmental sustainability principles. In addition, AOPs treatment systems can also be designed to operate in situ, in controlled environments, like in closed or semi-closed systems.

Some studies have tested the use of AOPs for the degradation of PST in freshwater and water treatment plants [47,80]. However, the results could be completely different in saltwater/seawater, as parameters like pH, organic matter and the presence of salts will affect reaction mechanisms and efficiency [47,48]. The only known study applying AOPs to the degradation of PST and PST-producing algae is quite recent [46]. The authors used micro-nanobubble (MNB)-assisted AOPs to inactivate *G. catenatum* cells and evaluate the fate of the released PST. Among the tested combinations—O_3_, O_3_/H_2_O_2_, and O_3_/H_2_O_2_/UV, with and without MNBs, they observed that the O_3_/H_2_O_2_/UV/MNBs combination was able to completely inactivate *G. catenatum* cells after 6 min, keeping cell membrane integrity. Also, they reported a 98.17% decrease in the overall toxicity of PST (C1, C2, GTX3, dcGTX2, dcGTX3) after 6 min, attributing this efficacy to the strong oxidative potential of the OH radical [46]. These results and the underlying mechanism of action of AOPs indicate that these methods could be effective in degrading PST and reducing toxicity within minutes. Further tests need to be conducted with bivalves in seawater to demonstrate the effectiveness of AOPs in a short period of time. However, if the prospects are confirmed, this could be a rapid and efficient approach to bivalve detoxification. Nevertheless, the implications of the AOPs costs cannot be overlooked. Initial investment, technical skills, as well as continuous operational and maintenance expenses associated with these advanced technologies may present an obstacle to general adoption, particularly in developing regions. Further studies and scaled-up trials are required to fully assess the technical and economic feasibility of these emerging processes, as well as their environmental impacts.

### 2.3. Comparative Analysis of the Various Removal Technologies

Different removal methods for PST have been analyzed to date, each with its advantages and disadvantages. Table 1 presents a summary of the strengths and limitations of the methods mentioned and proposes some recommendations for improvement.

The primary limitations of the detoxification methods described in the literature are related to their scalability and the challenges of replicating laboratory performance in field trials. Additionally, the structural and environmental complexity of PST significantly affects the application and efficacy of these methods, given the interconversion of toxins as well as the competition between toxin molecules and other components of the aquatic ecosystem for binding sites. Moreover, implementation costs and the lack of knowledge regarding the environmental implications of most of these methods call for further and deeper studies. In fact, to the best of our knowledge there are no studies on the life cycle assessment (LCA) of potential solutions or products applied to bivalve detoxification, while the issue of environmental sustainability is rarely addressed, and only very superficially. This is a major flaw that hinders policymaking by decision-makers and may diminish the interest of potential stakeholders in investing in the development of detoxification methodologies. It is therefore urgent to move toward these studies, also considering the environmental, economic, and social impacts of implementing the measure, using a life cycle sustainability assessment (LCSA) approach. This is a relevant tool that supports decision-makers so that potential solutions can be consciously implemented in real-world settings. For example, in our view, LCSA methods that require significant technical expertise and high energy costs will differ from those that use biodegradable or reusable materials, but specific studies that delve deeper into these issues are needed.

## 3. Conclusions and Perspectives

PST are toxic neurological compounds that induce PSP from contaminated seafood consumption. These toxins threaten our health, safety, and food security. Improper detoxification could lead to an outbreak of foodborne illness and death tolls owing to the lack of medications for conditions such as PSP. As a result, maximum efforts are required to contribute to the development of methods to remove them from bivalves. Until now, no method has been widely accepted for removing PST in bivalve and aquatic ecosystems. The structural complexity, biotransformation and interactions of these molecules in their environment also complicate the development of a generalized solution for PST removal. Advancing research on PST detoxification is not a choice but a necessity, especially given the high demand for seafood as a healthy and sustainable protein source. Investment in monitoring systems, design of removal technologies and analytical method development is paramount. The design and implementation of such technologies will boost regulatory compliance, foster consumer confidence, and bring about economic benefits. Collaboration between more developed and less developed countries for the global adoption of legal thresholds is essential for enhancing food safety, protecting public health, and ensuring equitable economic opportunities.

Various removal strategies have been proposed for the detoxification of PST from bivalves, each with its advantages and disadvantages. Harvest bans as a preventive measure are not sustainable, especially because of socio-economic losses and the continued demand for seafood. Owing to high capital loss and food security issues, this approach needs to be reviewed and adjusted to facilitate the development of a more lasting solution. Natural depuration and feeding procedures are green techniques for detoxifying bivalves but are not kinetically suitable. Industrial procedures using microwave systems or high heating procedures may reduce the quality and taste of bivalves. Additionally, the cost of running such a process is always a major concern, as far as energy is concerned. The adsorption process has been the most promising method, successfully balancing key factors such as cost-effectiveness, detoxification rate, and sustainability. This is especially due to its ability to modify adsorbents to more selectively suit their intended purpose and be recycled for reuse. Furthermore, adsorption offers a unique opportunity in terms of generating adsorbent materials from various biomasses, thereby reducing the cost of implementation while ensuring circularity. However, this procedure has certain challenges, such as the interference of other constituents of water. The type and structure of the predominant analogues, adsorbent pore size, and surface structure are some of the parameters that influence the efficiency and rate of detoxification by this process. Nevertheless, improvements can still be made by employing nanotechnology, for example, and combining the adsorption procedure with other potential methods. Additionally, further research into the surface modification of adsorbents, and optimization of the current results could bring us closer to the most suitable conditions and parameters for achieving a highly effective adsorption process for removing PST from bivalves. In turn, AOPs are emerging technologies in PST degradation that appear to have great potential. However, there are still many limitations in application and a long way to go.

Extensive research on these methods is crucial to verify their environmental impact and assess their future sustainability. Extending the research to field studies is also important. The costs associated with detoxifying bivalves can be substantial, particularly if the process is prolonged. The economic viability of these methods is a significant concern, as they must be cost-effective for adoption by the food industry. Thus, the cost, efficiency, kinetics, and quality of the bivalves should always be considered when selecting a method. In short, it is important to advance on several complementary fronts: (1) optimizing and scaling methods for detoxifying PST from bivalves; (2) developing ecotoxicological studies that evaluate potential by-products and ensure animal and human welfare; (3) assessing the sustainability (LCSA) of the entire process. When these conditions are met, decision-makers will have the basis to advance potential regulatory policies and mitigate existing flaws, ensuring greater food safety and reduced economic impacts.

## Figures and Tables

**Table 1:** Potential bivalve detoxification methods; strengths, limitations and recommendations for potential improvement.

Methods	Strengths	Limitations	Recommendations
Industrial and thermal procedures	Includes the only legally approved method for detoxifying the cockle *Acanthocardia tuberculata*. Simple, and widely used in food processing.	The approved method is very limited in terms of the number of species and toxins concentration. Not suitable for live shellfish detoxification. It alters the texture and marketability of seafood. More conventional heat treatments show little, or no efficiency. Energy costs.	Modify thermal treatment parameters to minimize nutrient loss. Explore low-energy microwave-assisted techniques. Combine heat treatment with other methods (e.g., adsorption) for better efficiency.
(Natural) depuration	Natural process. Suitable for live bivalves. It can be accelerated by the presence of non-toxic food.	It takes weeks to months depending on several factors such as species, toxin concentration, environmental conditions, etc. Increase in excreted by-products resulting from food supply.	Combine with other methods, such as adsorption, photodegradation or photocatalysis, to accelerate the detoxification process.
Adsorption	Suitable for live bivalves Conventional adsorbents (carbon- and chitosan-based, clays, resins) are relatively cheap and easy to obtain. Possibility of modifying the starting material. Removal efficiency demonstrated with different PST concentrations and bivalve species, within days (for most of the adsorbents). Ease of removal from the aqueous system (mainly for magnetic particles). Possible reuse.	Adsorbents removal from the system. The accumulation of nanoparticles in the environment raises potential eco-toxicological concerns. Efficiency varies with the type and properties of the adsorbent. Some modifiers may render the adsorbent more expensive and less environmentally sustainable. Efficiency may be reduced in high amounts of NOM and salts. The detoxification time is still longer than desirable (around 48 h [58]). Scarce toxicity studies for marine organisms.	Utilizing surface-modified clays can significantly improve toxin binding, while hybrid approaches offer promising synergistic effects. Environmental safety assessments should be conducted before large-scale applications to address potential eco-toxicological risks. Exploring bio-based nanomaterials presents a sustainable alternative. Improving the recovery and reuse of magnetic nanoparticles can further reduce operational costs.
Photodegradation and photocatalysis	Can rely on irradiation systems already installed in controlled systems (e.g., in purification centres). Relatively low cost. Does not require very specific technical skills. Photodegradation can be sensitized by DOM.	It is a complementary method of degrading toxins in an aqueous medium. It may result in the formation of byproducts. There are no known studies on seawater. Its effectiveness may be affected by the presence of naturally occurring substances.	Studies using seawater. Ecotoxicity studies of by-products. Combination with a priori techniques, particularly adsorption, to increase their combined effectiveness. Conduct field studies to assess real-world applicability.
AOPs	Fast toxin degradation, compared to other methods (within minutes). Suitable for live bivalves.	High operational and maintenance costs. Requires technical expertise. No studies were reported in seawater or involving bivalves. Its effectiveness may be affected by the presence of naturally occurring substances. Potential risks of toxic byproducts from oxidation reactions. The organoleptic properties of bivalves may be compromised due to non-selective oxidation by free radicals.	Develop cost-effective catalysts for sustainable AOPs. Development of studies using bivalves in seawater. Evaluation of bivalve properties after treatment. Ecotoxicity studies of by-products.

## Data Availability

Not applicable.

## Abbreviations

The following abbreviations are used in this manuscript:

AC	Activated carbon/charcoal
AOPs	Advanced Oxidation Processes
ASP	Amnesic Shellfish Poisoning
AZP	Azaspiracid Poisoning
BTAB	4,4′-bis(tributylammoniummethyl chloride)biphenyl
C1&2	N-sulfocarbamoyl Gonyautoxins 2 & 3
CMC	Carboxyl Methyl Chitosan
dcGTX2&3	Decarbamoylgonyautoxins 2 & 3
dcNEO	Decarbamoylneosaxitoxin
dcSTX	Decarbamoylsaxitoxin
DOM	Dissolved organic matter
DSP	Diarrheal Shellfish Poisoning
GTX1&4	Gonyautoxins 1 & 4
GTX2&3	Gonyautoxins 2 & 3
GTX5	Gonyautoxin 5
GTX6	Gonyautoxin 6
HAB	Harmful Algal Blooms
LCA	Life Cycle Assessment
LCSA	Life Cycle Sustainability Assessment
MC	Modified clay
MNBs	Micro-Nanobubbles
NOM	Natural Organic Matter
NSP	Neurotoxic Shellfish Poisoning
PAC-MC	Polyaluminium Chloride-Modified Clay
PMPS-MC	Peroxymonosulfate modified clay
PSP	Paralytic Shellfish Poisoning
PST	Paralytic shellfish toxins
SiO2-MA-CS	Silica-malic acid chitosan hydrogel
STX	Saxitoxin
TBTA	4-(tributylammoniummethyl)-benzyltributylammonium chloride
WAC	Wood-based activated carbon
